# Aptamer-mediated impairment of EGFR-integrin αvβ3 complex inhibits vasculogenic mimicry and growth of triple-negative breast cancers

**DOI:** 10.1038/srep46659

**Published:** 2017-04-20

**Authors:** Simona Camorani, Elvira Crescenzi, Matteo Gramanzini, Monica Fedele, Antonella Zannetti, Laura Cerchia

**Affiliations:** 1 Istituto per l’Endocrinologia e l’Oncologia Sperimentale “G. Salvatore” (IEOS), Consiglio Nazionale delle Ricerche (CNR), Via S. Pansini 5, 80131 Naples, Italy; 2 Istituto di Biostrutture e Bioimmagini (IBB), Consiglio Nazionale delle Ricerche (CNR), Via T. De Amicis 95, 80145 Naples, Italy

## Abstract

Current treatment options for triple-negative breast cancers (TNBCs) is limited by the absence of well-defined biomarkers, excluding a targeted therapy. Notably, epidermal growth factor receptor (EGFR) is overexpressed in a great proportion of TNBCs and is a negative prognostic factor. In clinical trials, however, existing EGFR inhibitors showed disappointing outcome. Oligonucleotide aptamers are a valid alternative to antibodies for diagnostic and therapeutic uses. Here, we prove that, when applied to aggressive TNBC cell lines with unique stem-like plasticity, the anti-EGFR CL4 aptamer, but not erlotinib or cetuximab, prevents the vasculogenic mimicry (VM) capability of the cells and destroys previously formed channels in three-dimensional culture. Notably, we found that CL4 impairs the matrix-induced integrin αvβ3 interaction with EGFR and integrin αvβ3-dependent cell adhesion. Consistently, the aptamer strongly inhibits VM and tumor growth in a xenograft TNBC model. These data suggest that in TNBC cells, EGFR may cooperate with integrin αvβ3 to regulate integrin binding to extracellular ligands required for VM, and EGFR-targeting by CL4 aptamer may counteract this event. Overall, we demonstrate a novel mechanism of action for CL4 related with integrin αvβ3-EGFR interaction, that may help to develop new oligonucleotide-based strategy addressing unmet need for TNBCs therapy.

Triple-negative breast cancers (TNBCs) account for ~15% of all breast cancers and approximately 170,000 patients worldwide are diagnosed annually with TNBCs^1^. TNBCs are a heterogeneous group of tumors consisting of different subtypes with unique biology and distinct clinical behavior^2^,^3^,^4^. Compared to other breast cancers, TNBCs usually affect younger patients, are larger in size, of higher grade and biologically more aggressive^1^. TNBCs are characterized by the absence of estrogen receptor, progesterone receptor and ErbB2, excluding the possibility of using efficacious targeted therapies developed against these proteins^5^. Thus, chemotherapy is the only way to treat a TNBC. However, even though having higher rates of clinical response to neoadjuvant chemotherapy, TNBC patients show high risk of recurrence and visceral metastasis and their death rate is disproportionately higher than any other subtype of breast cancer^6^. Despite tremendous effort has been devoted over the last few decades in searching effective targeted therapy, management of TNBCs is still challenging.

It has been recently shown that cells of aggressive and poorly differentiated TNBCs have the capability to undergo endothelial trans-differentiation thus forming vessel-like networks that provide blood supply for tumor growth and significantly increase cancer cells transfer thus promoting metastasis^7^,^8^,^9^,^10^. Consequently, hampering this phenomenon, known as vasculogenic mimicry (VM), may play a crucial role in a successful treatment of TNBCs.

Importantly, TNBCs often express genes that are characteristic of basal epithelial cells, including epidermal growth factor receptor (EGFR)^11^. While EGFR mutations are rare in TNBCs, high EGFR copy number is relatively frequent, correlates with EGFR overexpression that has been reported in ~60% of TNBCs and is associated with poor outcome^12^, thus indicating EGFR as a clinically relevant target in TNBCs. However, preclinical studies reveal that most TNBC cell lines are relatively resistant to EGFR inhibitors, as single agents^11^,^13^. Accordingly, therapies targeting EGFR with tyrosine kinase inhibitors (TKIs)^14^,^15^,^16^ or monoclonal antibodies (MAbs)^17^, while showing efficacy in other tumors, have not delivered long term benefits to TNBCs patients. Although the reasons for the failure of the EGFR-targeted therapy are unclear, emerging evidences indicate complex interplays among signaling pathways with potential to confer resistance to EGFR inhibitors^18^,^19^. Thus, identifying new strategies for TNBCs resistant to conventional EGFR inhibitors may open new doors of treatments.

Highly selective compounds emerging for anti-cancer therapy are aptamers isolated by the Systematic Evolution of Ligands by EXponential enrichment (SELEX) process^20^,^21^. Aptamers are short, artificial, single-stranded oligonucleotides that, similarly to antibodies, interact at high affinity with their targets by recognizing a specific three-dimensional (3D) structure. They offer unique chemical and biological characteristics like small size, high stability, lack of immunogenicity and also ready synthesis and manipulation, that render them a valid alternative to antibodies as recognition elements for *in vivo* tumor imaging and therapy.

Aptamers have been used as cancer therapeutics because of their ability to inhibit their targets and, more recently, as carriers for cell-targeted delivery of therapeutic secondary reagents^22^,^23^.

We have generated a nuclease resistant RNA-aptamer, named CL4, that binds at high affinity to the extracellular domain IV of human EGFR and inhibits the receptor in non-small cell lung carcinoma (NSCLC) and glioblastoma (GBM)^24^,^25^,^26^. Further, CL4 aptamer has been recently shown to specifically deliver nanoparticles containing therapeutic anti-miRNA to orthotopic TNBC MDA-MB-231 tumors in nude mice^27^.

In this study, we prove that CL4 aptamer, by binding to EGFR, impairs the matrix-induced interaction of the receptor with integrin αvβ3 on membrane of TNBC cells, thus impeding integrin αvβ3-dependent cell adhesion and VM in 3D cell culture condition which, conversely, were not affected by erlotinib and cetuximab. Consistently, the aptamer inhibited EGFR-integrin αvβ3 interaction, VM and tumor growth in a xenograft TNBC model. Collectively, our findings suggest a novel function for EGFR as crucial player in αvβ3 integrin-mediated VM, and indicate the CL4 aptamer as a promising tool for new therapeutic intervention in EGFR-positive TNBCs.

## Results

### The anti-EGFR aptamer inhibits tube formation ability of mesenchymal-like TNBC cells

**Figure 1:**
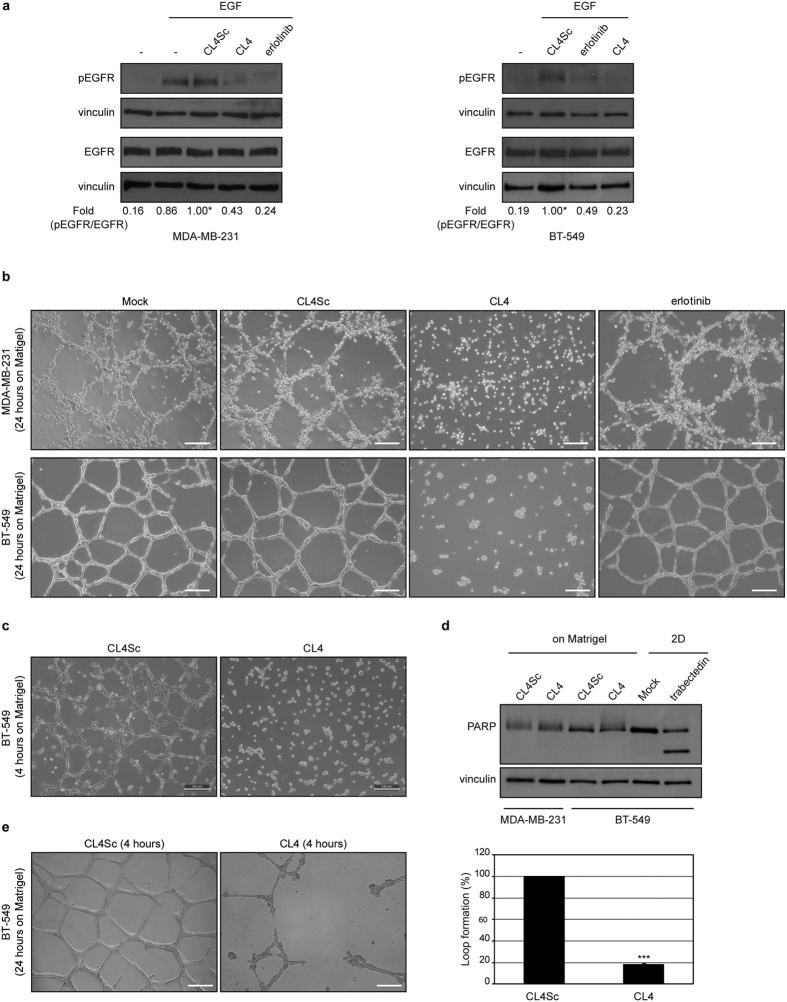
CL4 prevents TNBC cells ability to form VM channels on Matrigel and destroys preformed VM. (**a**) MDA-MB-231 and BT-549 cells were serum-starved for 18 hours and then left untreated or stimulated with 20 ng/ml EGF in the absence or in the presence of 10 μmol/l erlotinib or 200 nmol/l CL4 or CL4Sc, for 15 minutes, as indicated. Cell lysates were immunoblotted with anti-pEGFR and anti-EGFR antibodies. Equal loading was confirmed by immunoblot with anti-vinculin antibody. Values below the blot indicate the ratio of pEGFR to total EGFR signal levels, normalized to the respective vinculin signal level, and reported as relative to EGF stimulated cells in the presence of CL4Sc, arbitrarily set to 1 (labeled with asterisk). (**b**) MDA-MB-231 and BT-549 cells were seeded on Matrigel monolayer in the absence or in the presence of 200 nmol/l CL4 or CL4Sc or 10 μmol/l erlotinib for 24 hours. (**c**) BT-549 cells were seeded on Matrigel, in the presence of 200 nmol/l CL4 or CL4Sc aptamers, for 4 hours. Note that just 4 hours CL4-treatment is sufficient to prevent VM channels formation. (**d**) Lysates from MDA-MB-231 and BT-549 cells grown on Matrigel for 24 hours, in the presence of CL4Sc or CL4, as in (**b**), were immunoblotted with anti-PARP antibody. Vinculin was used as a loading control. The 24 hours-treatment of BT-549 cells with 10 nmol/l trabectedin, an antitumor drug, was used as a positive control of PARP cleavage. (**e**) BT-549 cells were grown on Matrigel for 24 hours and then treated with 200 nmol/l CL4 or CL4Sc for 4 hours. Destruction of preformed tubes was determined as the percentage of intact loops of CL4-treated cells compared with control. (**b,c,e**) Cells were photographed by phase-contrast microscopy. Representative photographs of at least five independent experiments were shown. Magnification 10×, scale bar = 200 μm. Bars depict means ± SD. ***P < 0.001 relative to CL4Sc-treatment.

The EGFR-positive BT-549 and MDA-MB-231 TNBC cell lines well reflect the undifferentiated mesenchymal-like (ML) subtype to which they have been assigned^2^. As shown (Supplementary Fig. S1), compared to other breast cancer cell lines, they share high expression levels of vimentin and undetectable E-cadherin, accordingly to their unique features of epithelial mesenchymal transition (EMT). Further, unique to these cell lines is the expression of the platelet-derived growth factor receptor β (PDGFRβ) that is emerging as a mesenchymal/stem cell-specific marker in different human cancers^2^,^28^ and a player of VM in TNBC^29^. In agreement with their remarkable degree of plasticity, both BT-549 and MDA-MB-231 cells began to form tubular structures in 4 hours when plated on Matrigel monolayer, a hallmark of VM, and formed very organized channels at longer times, up to 24 hours (Supplementary Fig. S2). Thus, we wondered whether the anti-EGFR CL4 aptamer could hamper the tube formation ability of the cells. For this purpose, first, we verified the neutralizing effect of the aptamer on EGFR activation in both TNBC cell lines. As shown (Fig. 1a), EGF stimulation, following serum starvation, significantly increased phosphorylation of EGFR and both the anti-EGFR aptamer and erlotinib TKI efficiently inhibited ligand-induced EGFR phosphorylation. A scrambled sequence (CL4Sc) was used as a negative control. Next, MDA-MB-231 and BT-549 cells were plated on Matrigel in the presence of CL4 (200 nmol/l-treatment) or erlotinib (10 μmol/l-treatment) and vascular-like network formation was assessed up to 24 hours. Notably, we found that the anti-EGFR aptamer strongly prevented the vasculogenic capacity of the cells at 24 hours, whereas erlotinib was not able to affect this phenomenon (Fig. 1b). Importantly, 4-hour CL4 treatment was sufficient to impede tube formation (Fig. 1c). No effect on cell viability was obtained up to 72 hours, by using the 200 nmol/l-dosage of aptamer as for the tube formation assay, that was renewed each 24 hours (Supplementary Fig. S3). Further, no poly (ADP-ribose) polymerase (PARP) cleavage was observed by Western blot analysis on lysates from CL4-treated cells harvested from Matrigel after 24 hours incubation (Fig. 1d), thus indicating that the aptamer effect on inhibition of channels formation was not due to inhibition of cell viability neither to apoptosis. Next, to determine whether the aptamer is able not only to prevent tube formation but also to destroy the previously formed channels, BT-549 cells were grown on Matrigel for 24 hours to form VM channels and then treated with 200 nmol/l CL4 or CL4Sc for 4 hours. As shown in Fig. 1e, CL4 caused the elimination of the web-like structure that remained intact after treatment with CL4Sc negative control.

Taken together, these results indicate that aptamer-mediated EGFR inhibition prevents TNBC cells organization into vessel-like structures on Matrigel monolayer and destroys preformed VM channels.

### The anti-EGFR aptamer blocks the endothelial trans-differentiation of TNBC cells

**Figure 2:**
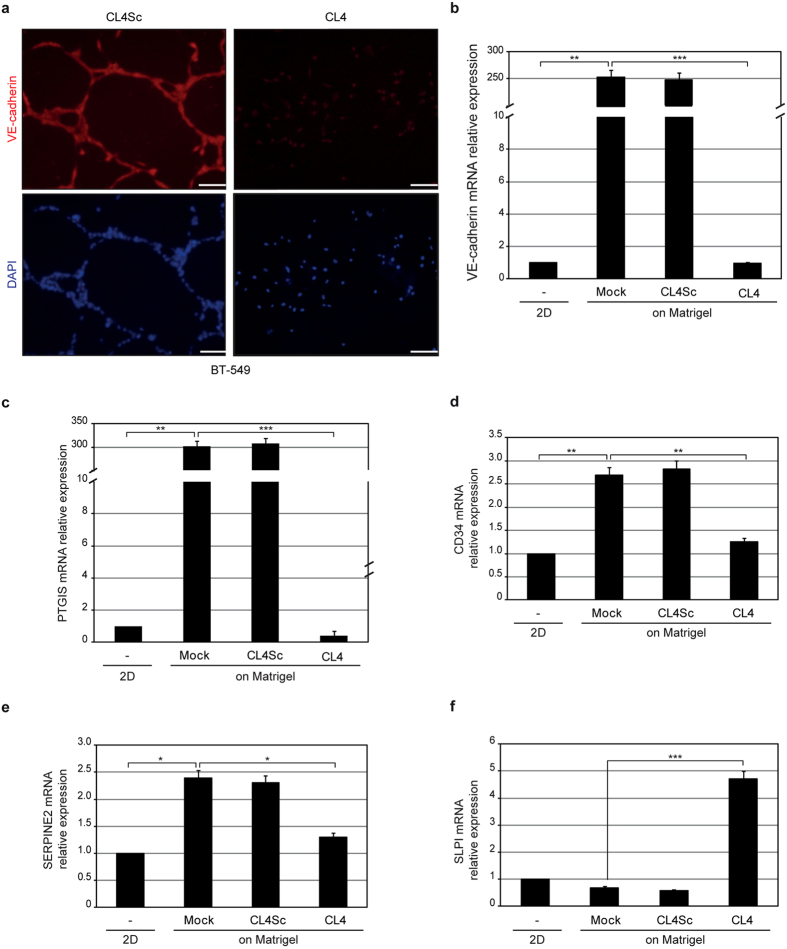
The anti-EGFR aptamer blocks the endothelial trans-differentiation of BT-549 cells. (**a**) BT-549 cells, seeded on Matrigel in the presence of 200 nmol/l CL4 or CL4Sc for 24 hours, were stained with anti-VE-cadherin antibody, visualized by fluorescence microscopy and photographed; nuclei were stained with DAPI. Magnification 20×, scale bar = 100 μm. (**b–f**) BT-549 cells were left untreated or treated as in (**a**) and mRNA levels of the indicated VM-genes were determined by RT-qPCR. Bars depict means ± SD of three independent experiments. ***P < 0.001; **P < 0.01; *P < 0.05; one-way ANOVA followed by Tukey’s multiple comparison test. No statistically significant variations among CL4Sc- and mock-treatment was obtained.

In VM, tumor cells assume endothelial function and form vessel-like structures. To assess the effect of the aptamer on TNBC cells trans-differentiation into endothelial-like cells, BT-549 cells were seeded on Matrigel-coated coverslip in the presence of CL4 or CL4Sc negative control and the expression of the vascular endothelial cadherin (VE-cadherin) was evaluated by fluorescence microscopy at 24 hours after plating. As shown (Fig. 2a), cells grown on Matrigel acquired endothelial behavior with VE-cadherin expression. However, in the presence of CL4 aptamer very low amount of VE-cadherin staining was observed thus confirming the aptamer ability to prevent VM channels. To further support these findings, the change in the expression of VM-genes in the presence of aptamer treatment was monitored by RT-qPCR on mRNA extracted from BT-549 cells grown on Matrigel for 24 hours. As shown (Fig. 2b), when seeded on Matrigel, cells strongly upregulated the VE-cadherin gene, in agreement with fluorescence-microscopy analyses, and endothelial cell markers Prostaglandin I-2 synthase (PTGIS) (Fig. 2c) and CD34 (Fig. 2d), thus confirming their capacity to trans-differentiate into endothelial cells. Accordingly with the above findings, CL4, but not CL4Sc negative control, strongly inhibited VM-genes expression (Fig. 2b–d). Also, CL4 counteracted the induction of expression of the serine protease inhibitor (SERPINE2) (Fig. 2e), a gene reported to be amplified in tumors with marked VM^30^ and recently shown to be crucial in programming aggressive breast cancer cells for VM^9^. Conversely, in the presence of CL4 treatment we observed a significant upregulation of secretory leukocyte protease inhibitor (SLPI) expression (Fig. 2f), accordingly with its described role as a suppressor of tumor growth and metastasis in breast cancer^31^,^32^.

In summary, the CL4-mediated impairment of tube formation is accompanied by important changes in expression of VM-related genes.

### The anti-EGFR aptamer impairs Matrigel-induced EGFR-integrin αvβ3 complex

During canonical angiogenesis as well as VM process, vessel formation is essentially dependent on cell-matrix interaction. Integrins are cell surface adhesion molecules representing the main receptors by which the cells bind to and respond to extracellular matrix (ECM) components^33^,^34^. Among them, integrin αvβ3 expression strongly correlates with tumor invasion, EMT and metastases of highly aggressive cancers^33^,^34^,^35^ and contributes to channel-like structures formation of human melanoma cells^36^. Importantly, multiple receptor tyrosine kinases (RTKs) have been shown to associate with integrin αvβ3, thus promoting many aspects of tumor progression^37^. In particular, in the presence of ECM components, integrin αvβ3 forms a complex with EGFR and EGFRvIII mutant thus inducing motility of MDA-MB-231 and GBM cells, respectively^38^,^39^. Further, in response to cell matrix adhesion, integrin αvβ3 associates with EGFR in a macromolecular complex on the surface of human ECV304 and EGFR-transfected NIH3T3 cells^40^.

**Figure 3:**
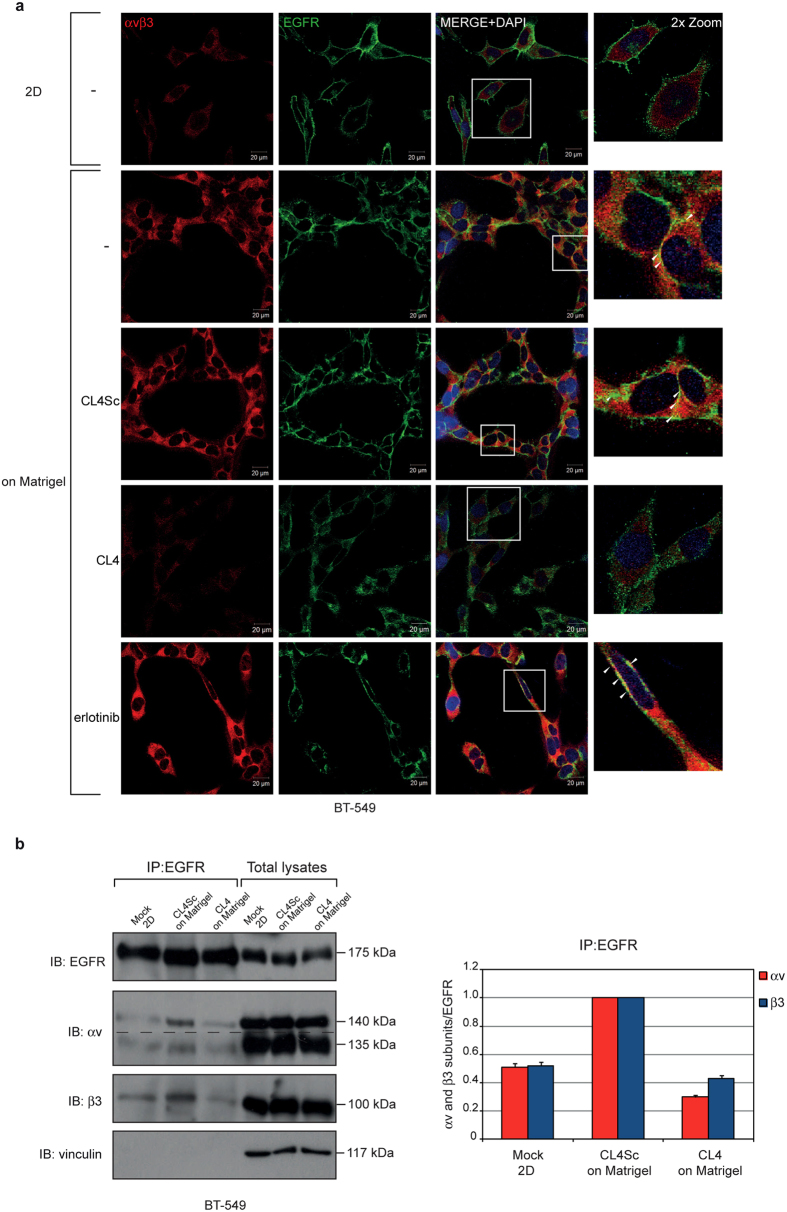
CL4 impairs Matrigel-induced EGFR-integrin αvβ3 interaction. (**a**) BT-549 cells grown in 2D or on Matrigel in the absence or in the presence of 200 nmol/l CL4 or CL4Sc or 10 μmol/l erlotinib for 24 hours were fixed, permeabilized and labelled with anti-αvβ3 LM609 (red) and anti-EGFR (green) antibodies. Co-localization results appear yellow in the merged images. Nuclei were stained with DAPI. All digital images were captured at the same setting to allow direct comparison of staining patterns (Magnification 63×, 0.7× digital zoom). Scale bar = 20 μm. White square indicate the area showed in insets. Arrowheads indicate some co-localization points between EGFR and integrin αvβ3. (**b**) Equal amounts of lysates from BT-549 cells grown in 2D or on Matrigel in the presence of 200 nmol/l CL4 or CL4Sc were directly subjected to Western blotting or prior immunoprecipitated with anti-EGFR antibody. Filter was cut in two pieces that were immunoblotted with anti-EGFR and anti-integrin β3 antibodies, stripped, rejoined and immunoblotted with anti-integrin αv antibody. Dashed line indicates the boundary between the two pieces of the filter. Vinculin was used as a loading control. Molecular weights of indicated proteins are reported (*left*). The bands were quantified by densitometric analysis and the amount of αv or β3 co-immunoprecipitated with EGFR relative to immunoprecipitated EGFR levels is reported. Values are shown relative to CL4Sc control, arbitrarily set to 1 (*right*). The data shown here represent three experiments exhibiting similar effects. Note that no significant change in αv, β3 and EGFR levels was observed in total lysates following CL4 treatment.

Thus, we analyzed tumor cells growing on Matrigel monolayer for a possible co-localization between EGFR and integrin αvβ3. To this aim, BT-549 cells were immunostained for EGFR and integrin αvβ3 and analysed by confocal microscopy. For integrin αvβ3 staining we used the anti-αvβ3 LM609 monoclonal antibody which binds to a conformational epitope resulting from the association of the αv and β3 subunits. As shown, when cells were seeded on Matrigel, puncta of co-localization between integrin αvβ3 and EGFR were appreciable on the cell membrane (Fig. 3a). Interestingly, in the presence of CL4 treatment, integrin αvβ3-staining, that appeared of diminished intensity with respect to control cells (untreated and CL4Sc-treated), failed to co-localize with EGFR-staining, thus resembling the situation observed in 2D classical grow (Fig. 3a). Conversely, a clear co-localization between EGFR and integrin αvβ3-associated signals was still observed in the presence of erlotinib (Fig. 3a). Further, in order to exclude cytoplasmic staining, we performed immunofluorescence analyses on un-permeabilized cells, grown on Matrigel in the absence and in the presence of CL4 treatment. As shown, multiple yellow dots were accumulated on the membrane of control cells and very little to no signal was revealed on CL4-treated cells (Supplementary Fig. S4). These findings suggest that in Matrigel αvβ3 forms a complex with EGFR on cell membrane and that CL4 interferes with such complex. In order to verify this hypothesis, we performed co-immunoprecipitation assays, revealing that, when cells were seeded on Matrigel, the integrin αvβ3 subunits immunoprecipitated with EGFR at an extent 2.0 (αv subunit) and 1.9 (β3 subunit) times higher than in 2D (Fig. 3b). We verified that αv and β3 subunits were in complex while interacting with EGFR by performing immunoprecipitation of cell lysates with LM609 antibody recognizing the αvβ3 integrin complex, followed by immunoblotting with anti-EGFR antibody (Supplementary Fig. S5). Importantly, CL4 caused a reduction of integrin subunits levels bound to EGFR of about 3.4 (αv subunit) and 2.3 (β3 subunit) times (Fig. 3b).

No change in the protein levels of integrin αv and β3 subunits following CL4 treatment was observed by Western blot analyses performed on total cell lysates (Fig. 3b), thus indicating that the apparent reduction of the integrin αvβ3 immunofluorescent staining in CL4-treated cells, as observed in Fig. 3a, was not due to integrin downregulation. It is likely that the aptamer, by interfering with EGFR-integrin αvβ3 interaction, affects the integrin binding to the anti-αvβ3 LM609 antibody thus reducing the staining intensity.

Similarly, immunoprecipitation analyses showed integrin αvβ3-EGFR interaction in MDA-MB-231 cells on Matrigel monolayer (Supplementary Fig. S6).

These data indicate that in response to matrix, integrin αvβ3 associates with EGFR on cell surface and adopts a conformation crucial for VM formation. CL4 dissociates αvβ3 integrin from EGFR without affecting their expression and, plausibly, affects integrin αvβ3 binding to matrix ligands and VM.

### The anti-EGFR aptamer inhibits integrin αvβ3-dependent cell adhesion

**Figure 4:**
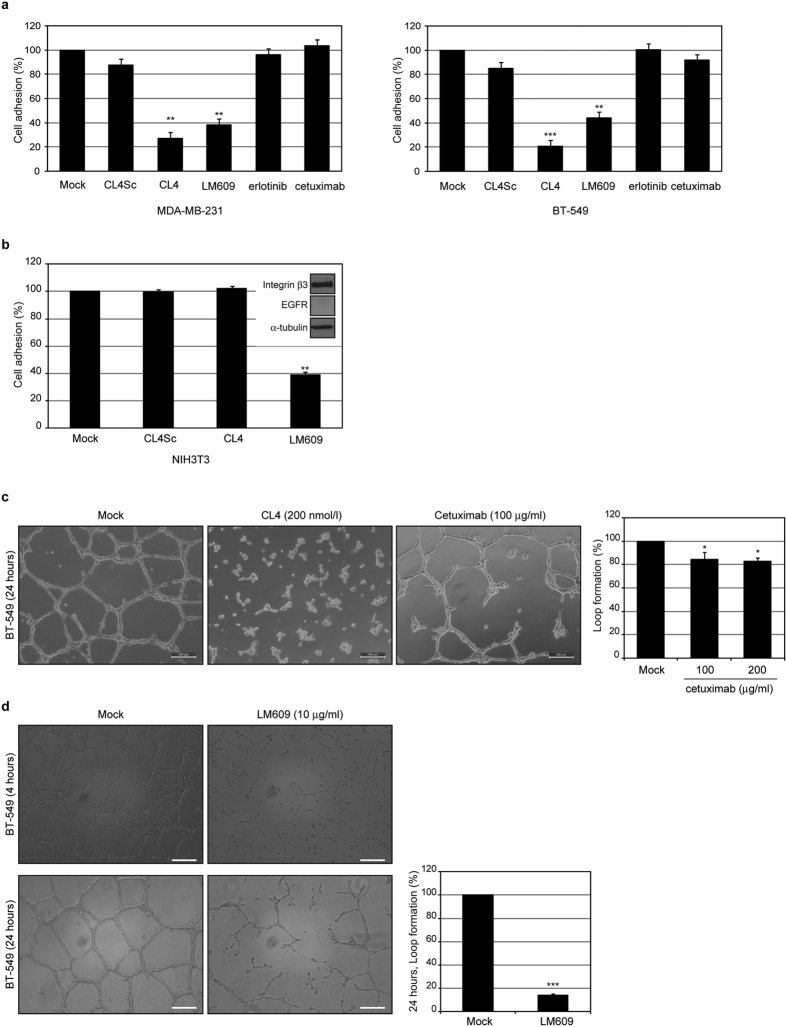
CL4 inhibits αvβ3-dependent cell adhesion to vitronectin. (**a,b**) Cells were mock-treated or pretreated with 200 nmol/l CL4 or CL4Sc, 10 μmol/l erlotinib, 100 μmol/l cetuximab or 10 μg/ml anti-αvβ3 LM609, as indicated, and then subjected to the adhesion assay on vitronectin-coated plates. Results are expressed as percentage of adherent cells considering the mock-treated control cells as 100%. In **b** (insert), lysates from NIH3T3 cells were immunoblotted with anti-integrin β3, anti-EGFR and α-tubulin antibodies. (**c,d**) Representative phase-contrast images of BT-549 cells grown on Matrigel monolayer for the indicated times in the absence or in the presence of 200 nmol/l CL4 or 100 μmol/l cetuximab (**c**) or 10 μg/ml anti-αvβ3 LM609 (**d**). Magnification 10×, scale bar = 200 μm. Tube formation ability was determined as the percentage of reduction in loop formation of treated cells compared with mock-treated cells. Note that in the presence of CL4 treatment no loops were observed. Bars depict means ± SD of three independent experiments. ***P <* *0.001; **P <* *0.01; *P <* *0.05 relative to mock-treated cells; one-way ANOVA followed by Tukey’s multiple comparison test (**a–c**) or student’s *t*-test (**d**).

To confirm that upon CL4 impairment of integrin αvβ3-EGFR interaction, integrin αvβ3 is no longer competent for binding to matrix, we performed adhesion assays of MDA-MB-231 and BT-549 cells to vitronectin, the major ECM ligand of integrin αvβ3. As shown (Fig. 4a), pre-treating the cells for 1 hour with CL4, caused a dramatic reduction of their adhesion to vitronectin as compared with controls treatment (mock and CL4Sc), and comparable to that reached in the presence of the anti-αvβ3 LM609 blocking antibody, used as a positive control. Further, in agreement with the above findings, erlotinib-treatment did not elicit any effect on cells adhesion (Fig. 4a). Next, to confirm that the aptamer effect is dependent on EGFR expression, we verified that CL4 does not interfere with adhesion capability of EGFR-negative NIH3T3 cells (Fig. 4b), that have been reported to adhere to vitronectin via integrins^41^. Recently, cetuximab, an EGFR-targeting monoclonal antibody, has shown a modest survival benefit in patient with metastatic TNBC, used in combination therapy with cisplatin^42^. Even if cetuximab has been reported to poorly affect proliferation of TNBC cell lines in 2D classical cultures^13^, we assessed whether by binding to the ectodomain of EGFR it is able to exert a neutralizing effect on TNBC cell adhesion to ECM, as the anti-EGFR aptamer. Notably, despite the antibody inhibited EGF-stimulated phosphorylation of EGFR in both MDA-MB-231 and BT-549 cells (Supplementary Fig. S7), it did not cause an appreciable inhibition of cell adhesion to vitronectin (Fig. 4a) even at a concentration (100 μg/ml) higher than steady state plasma levels in cancer patients administered with the clinical dosing regimen^43^. However, a little effect on TNBC tube formation (much lower than that produced by CL4) was observed in the presence of 100 μg/ml cetuximab treatment, and did not increase any further at higher concentration (Fig. 4c). Whether the different behavior of the aptamer and the antibody is due to the different epitopes on the receptor recognized by the two EGFR inhibitors, remains to be determined.

Next, to confirm the direct link between the VM program and the biological function of αvβ3 integrin, we performed the tube formation assay in the presence of the anti-integrin αvβ3 blocking antibody. In agreement with what reported for human melanoma cells^36^, the LM609 antibody significantly inhibited the vasculogenic capacity of BT-549 cells (Fig. 4d), thus supporting that the conformational change of αvβ3 is the main mechanism of CL4-mediated VM inhibition.

These data suggest a crucial role for EGFR in modulating integrin αvβ3-mediated processes required for cell adhesion to ECM and tube formation and indicate the CL4 aptamer as an important tool to interfere with such processes.

### The anti-EGFR aptamer inhibits tumor growth and VM *in vivo*

**Figure 5:**
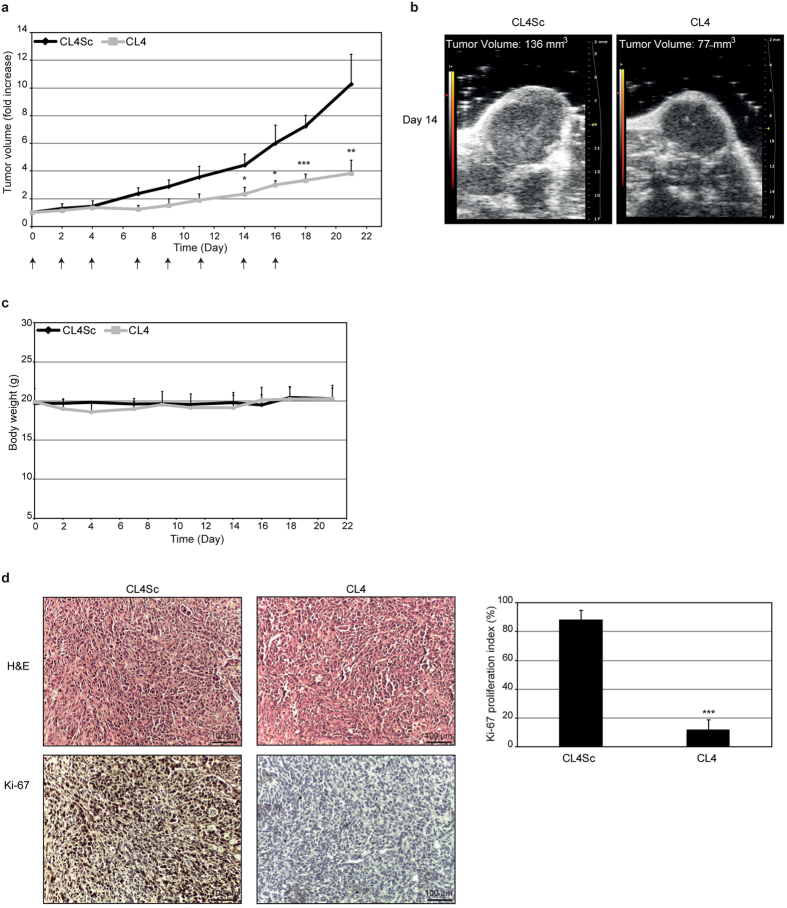
CL4 inhibits tumor growth. (**a**) Mice bearing MDA-MB-231 xenografts were injected intravenously with CL4 or CL4Sc, used as a negative control, at the times indicated by the arrows. Day 0 marks the start of treatments. Tumor growth was monitored by calipers over times and experimental raw data (expressed as fold increase) were interpolated with no curve fitting or regression analysis. ***P < 0.001; **P < 0.01; *P < 0.05 relative to CL4Sc (n = 6). (**b**) Tumor volume was measured by high-frequency ultrasound at day 14. Example shows analysis in one representative animal from each treatment group. (**c**) Mice body weight was measured at the indicated days and the mean weight of each group is shown. (**d**) Representative sections of tumors from CL4Sc and CL4 groups were stained with H&E (upper panels) and Ki-67 (lower panels) antibody. Ki-67 proliferation index was calculated as the percentage of Ki-67 positive cells/total cell count for 10 randomly selected 40× microscopic fields (n = 3 individual tumors, ***P < 0.001 relative to CL4Sc). Magnification 20×, scale bar = 100 μm. In (**a**,**c**,**d**) error bars depict means ± SD.

We next investigated the effect of CL4 aptamer on tumor growth and VM formation in MDA-MB-231-derived mouse xenografts. To this aim, CL4 and CL4Sc, used as a negative control, were intravenously administered (1.04 mg aptamer/kg) at day 0, 2, 4, 7, 9, 11, 14, 16. Tumor growth was monitored by calipers (Fig. 5a) over time (up to 21 days) and, when the difference of tumor growth between CL4Sc and CL4 treatments became statistically significant, e.g. at day 14 (4.3 ± 1.13 *vs* 2.53 ± 0.74, respectively *P* = 0.02), the tumor growth was also monitored by high-frequency ultrasound (Fig. 5b) to confirm response to treatment. As shown, tumors were reduced significantly in CL4-treated mice compared with control CL4Sc-treated group. The treatments did not decrease the tumor-bearing mice body weight thus indicating the absence of aptamer toxicity (Fig. 5c). The antitumor activity of CL4 was also confirmed by the strong reduction of the proportion of proliferating cells immunostained for Ki-67 with respect to CL4Sc (Fig. 5d). Accordingly to the anti-proliferative effect observed *in vivo*, the aptamer inhibited the growth of MDA-MB-231 cells cultured in Matrigel for 7 days. Indeed, as shown in Supplementary Fig. S8a, CL4 affected the development of colonies that were drastically fewer, smaller in size as compared with well-developed branching colonies observed in control cells (mock and CL4Sc). Moreover, growth curve experiments showed reduced growth rate of both TNBC cell lines, grown in classical 2D condition in the presence of CL4; the growth inhibition became significant starting from day 4 and reached about 72% (MDA-MB-231 cells) and 60% (BT-549 cells) at day 7 with respect to cells mock-treated or treated with CL4Sc (Supplementary Fig. S8b). Importantly, no significant effects on cell growth were elicited by erlotinib treatment, both in 2D and in 3D culture (Supplementary Fig. S8).

**Figure 6:**
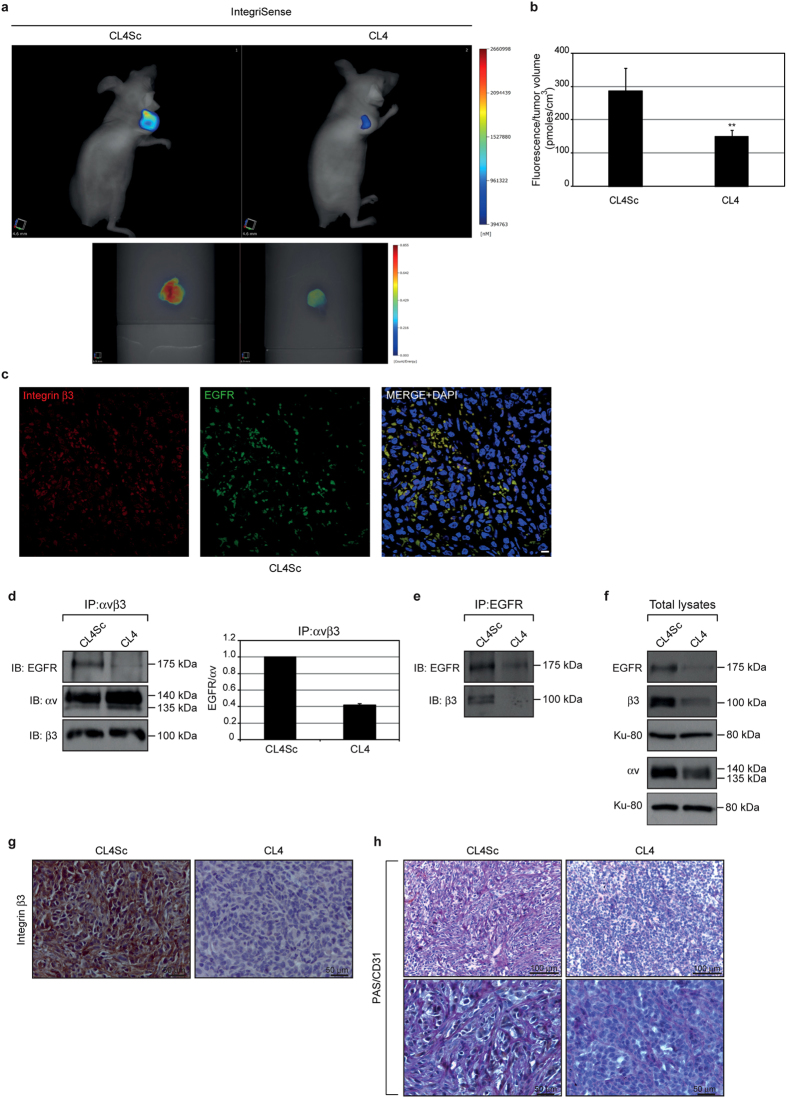
CL4 decreases IntegriSense signal in tumors and inhibits VM. (**a**,**b**) *In vivo* imaging and quantification of IntegriSense in tumor-bearing mice. (**a**) Representative volume renderings taken at the same color gating from CL4Sc- and CL4-treated mice injected with IntegriSense at day 21 (upper panels). Representative images of the single tumors excised from CL4Sc- and CL4-treated mice after *in vivo* imaging (lower panels). (**b**) The amount of fluorescence (pmoles) was quantified in specific ROIs encompassing the tumor in the animal and normalized to tumor volume (cm^3^). Error bars depict means ± SD. *P* = 0.0091 (n = 4). (**c**) Representative sections of tumors (CL4Sc group) were stained with anti-integrin β3 (red) and anti-EGFR (green) antibodies and analysed by confocal microscopy. Nuclei were stained with DAPI. Co-localization results appear yellow in the merged image. Magnification 63×, scale bar = 10 μm. (**d–f**) Equal amounts of lysates from recovered tumors were immunoprecipitated with anti-integrin αvβ3 LM609 antibody (**d**) or anti-EGFR antibody (**e**) and immunoblotted with the indicated antibodies. Total lysates were immunoblotted with anti-EGFR, anti-integrin αv and anti-integrin β3 antibodies, as indicated. Equal loading was confirmed by immunoblot with anti-Ku-80 antibody (**f**). Molecular weights of indicated proteins are reported. Representative data are shown from one of three independent experiments. In **d**, the histogram reports the amount of EGFR co-immunoprecipitated with integrin αvβ3 relative to αv levels. Values are shown relative to CL4Sc control, arbitrarily set to 1. (**g,h**) Representative sections of tumors harvested from CL4Sc and CL4 groups were stained with integrin β3 (**g**) and PAS/CD31 (**h**) as indicated. (Magnification 40×, scale bar = 50 μm; Magnification 20×, scale bar = 100 μm).

Next, to corroborate the cell culture findings, indicating that CL4 drastically hampers integrin αvβ3 functionality, the IntegriSense imaging agent, a high affinity ligand for integrin αvβ3, was injected (2 nmoles) in MDA-MB-231 tumor-bearing mice after 21-day treatment and imaging studies were performed at 24 hours post-injection by fluorescence molecular tomography (FMT) (Fig. 6a). Importantly, as shown in Fig. 6b, a significant 48% decrease of the amount of IntegriSense signal, normalized to tumor volume, was observed in the tumors of CL4-treated mice as compared to control tumors (*P* = 0.0091).

Moreover, xenografts post-three-week aptamer treatment were analyzed for EGFR and integrin αvβ3 interaction. First, the colocalization of EGFR and integrin β3 was verified in the control tumors by immunofluorescence (Fig. 6c). Next, tumor lysates were immunoprecipitated with anti- αvβ3 LM609 antibody and immunoblotted with anti-EGFR antibody. In agreement with *in vitro* findings, EGFR immunoprecipitated with αvβ3 in lysates from control tumors and CL4 treatment strongly reduced the amount of EGFR complexed to integrin αvβ3 (Fig. 6d). Similar results were obtained by immunoprecipitation of tumor lysates with anti-EGFR antibody and immunoblotting with anti-β3 antibody (Fig. 6e) showing that no integrin β3 band was detected in immunoprecipitates of EGFR in CL4 tumor lysates.

Interestingly, while the short term treatment of cells grown on Matrigel with CL4 could dissociate αvβ3 from EGFR without affecting their expression, the downregulation of EGFR, β3 and αv expression occurred upon the long term treatment of MDA-MB-231 xenografts with the aptamer (Fig. 6f). Consistent with the above findings, immunohistochemical staining for integrin β3 subunit on tumor sections revealed a high proportion of positive cells in control tumors, that was drastically reduced in the tumors from CL4-mice group (Fig. 6g).

To confirm *in vivo* the VM formation obtained by cells cultured on Matrigel, tumor sections were analysed by PAS/CD31 staining, a commonly used method for identification of VM^9^,^44^. As shown (Fig. 6h), control tumors contained networks of interconnected PAS-positive CD31-negative loops with evidence of tumor cells stretching to form the tubular channel-like structures; this morphological aspect, characteristic of VM formation^9^, appeared dramatically altered in the tumors from CL4-treated mice.

Altogether, our data suggest that the αvβ3-EGFR crosstalk has crucial roles in TNBC growth and VM. Therefore, aptamer-mediated targeting of αvβ3-EGFR interaction represents an attractive strategy for developing novel anti-cancer therapeutics.

## Discussion

Gene expression analyses identified six distinct TNBC subtypes including two basal-like, an immunomodulatory, a mesenchymal-like, a mesenchymal stem-like (MSL), and a luminal androgen receptor subtype, the last being characterized by androgen receptor signaling. Gene ontologies of these subtypes suggest driver genes and pathways that predict response to therapy^2^. Accordingly, basal-like subtypes, showing enrichment of proliferation genes, have a better response to taxane-based therapies as compared to MSL, characterized by a stem-cell like expression profile^2^,^4^. Further, the ML and MSL groups share signaling genes with the metaplastic breast cancer, a highly dedifferentiated neoplasm characterized by both epithelial and mesenchymal elements and high chemo-resistance^2^. Importantly, a large panel of human TNBC cell lines was established to be representative of these subtypes based on gene expression profiles and has been used as model to facilitate preclinical experiments aimed to define drug sensitivity of the distinct subtypes within this heterogeneous disease^2^,^3^. Among these cell lines, BT-549 and MDA-MB-231 cells largely recapitulate the gene expression patterns and mutations found *in vivo* and reflect the biological behavior of the ML and the MSL subtypes, to which they have been respectively assigned^2^,^45^. As shown in previous reports^29^,^46^,^47^,^48^ and herein, the two cell lines are characterized by the expression of EMT markers, highly malignant and invasive phenotype and a strong tendency to form VM channels both *in vitro* and *in vivo*. Both these cell lines, as most TNBCs and TNBC cell lines, express EGFR, nevertheless, they are extremely resistant to EGFR inhibitors^11^,^13^. It has become increasingly clear that the plasticity of TNBCs with a mesenchymal stem-like phenotype is pivotal in malignant tumor progression and VM, and may also in part account for the resistance to EGFR TKIs^18^. As a unique perfusion way, VM is associated with metastatic progression, drug resistance and poor patient prognosis^9^,^49^. Hence, a great challenge is the finding of innovative strategies able to counteract VM formation.

**Figure 7:**
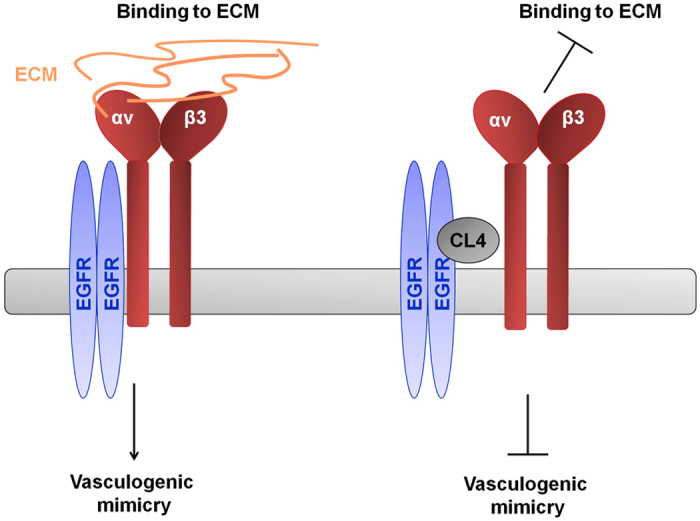
Proposed mechanism of action for CL4 aptamer related with integrin αvβ3-EGFR interaction. By binding to EGFR, CL4 aptamer impairs integrin αvβ3-EGFR interaction, causing inhibition of integrin binding to matrix and, in turn, VM.

In this study, we provide evidence that the anti-EGFR CL4 aptamer is able to inhibit BT-549 and MDA-MB-231 cells ability to form vascular-like structures when cultured on Matrigel, that resembles the *in vivo* extracellular matrix. The neutralizing effect on VM was largely recapitulated in MDA-MB-231 xenografts in nude mice, as revealed by immunohistochemical analyses on tumors. It has been shown that integrins expressed on tumor cells, e.g. αvβ3, are the major family of cell surface receptors that mediate attachment to ECM, being implicated in invasion, metastasis and VM of highly aggressive human cancers. Different examples of crosstalk between integrins and RTKs, also based on a physical interaction among them, have been reported to affect these properties^33^. Moreover, high expression of integrin αvβ3 has been recently shown as a marker of breast, lung and pancreatic carcinomas with stem-like properties that are highly resistant to TKIs erlotinib and lapatinib^18^. Importantly, we found that when TNBC cells are grown on Matrigel or subcutaneously injected in nude mice to form tumor, integrin αvβ3 is associated with EGFR and CL4 treatment impairs such a complex both *in vitro* and *in vivo*. Also, treating the cells with the aptamer drastically hampers the αvβ3-mediated cell attachment to vitronectin *in vitro* and strongly reduced the tumor uptake of the IntegriSense αvβ3-targeting agent, as assessed by *in vivo* imaging analyses of TNBC xenografts. Thus, we propose a new mechanism (Fig. 7) by which, in 3D TNBC culture environment and in TNBC xenografts, integrin αvβ3 is associated with EGFR on cell membrane; this interaction regulates integrin binding to extracellular ligands required for VM. Targeting EGFR, CL4 impairs integrin αvβ3-EGFR complex on cancer cells, causing inhibition of integrin binding to matrix and, in turn, VM.

Trastuzumab, a monoclonal antibody against epidermal growth factor receptor 2 (HER2), induces receptor internalization and degradation^50^ and reduces the growth of breast cancer xenografts with loss of integrin αvβ6 and HER2, existing in the same molecular complex on breast cancer cells^51^. Like Trastuzumab, CL4 has been previously shown to be able to actively internalize EGFR into the cells upon EGFR binding^26^ and, following long-term treatment of MDA-MB-231 xenografts, we here show that it causes a strong reduction in integrin αv/β3 subunits and EGFR levels in the tumors. Further studies will elucidate whether inhibiting the EGFR-integrin association in TNBC cells, the aptamer may elicit the receptors internalization and degradation. Several examples have been reported of crosstalk between integrins and RTKs that modify integrin traffic, determining degradation or recycling of the receptor, and regulate the engagement of matrix ligands and cancer cell behavior in 3D microenvironments^52^. Growth factor signals, by coupling MET and β1 integrin, stabilize MET by inhibiting its endocytosis^53^, it is therefore plausible that in TNBC cells the trafficking pathways of EGFR and αvβ3 integrin are strictly linked and the CL4 aptamer, by destabilizing EGFR-integrin complex, downregulates the levels of both proteins through their internalization and degradation.

In addition to the drastic inhibiting effect on VM program, we found that CL4 aptamer causes a strong inhibition of tumor growth, that is in agreement with its ability to inhibit TNBC cell proliferation in conventional 2D culture model. However, since VM contributes to feed the tumor it is likely that the drastic anti-proliferative effect observed is not only a direct consequence of CL4-mediated inhibition of EGFR activation, but also achieved through the reduction of VM. Moreover, it has been reported that the association of αvβ3 with certain RTKs (insulin receptors, PDGFRβ and vascular endothelial growth factor receptor 2, VEGFR2) in the presence of extracellular matrix ligands augments the ability of RTKs to respond to their growth factor ligands by inducing increased cell proliferation and migration^54^. Hence, given that integrin-RTK interactions result in a synergistic effect on their functions, the dual role of CL4 related to EGFR TKI inhibition and to impairment of αvβ3-EGFR complex may account for the drastic anti-proliferative and anti-VM effect observed *in vivo* and renders the aptamer an optimal candidate to be explored for TNBC treatment.

PDGFRβ and the VEGFR2 associate with integrin αvβ3 through the extracellular domain of the β3 subunit, independently from the RTKs phosphorylation, whereas the cytoplasmic and transmembrane regions of β3 subunit are dispensable^54^. It is plausible to speculate that the different mode of action of the aptamer and erlotinib may explain their different effects on TNBC cells. Indeed, the CL4 aptamer inhibits EGFR by binding to the receptor extracellular domain while erlotinib is an ATP competitive small molecule TKI. To this regard, the poor effect of cetuximab on inhibition of tube formation and cell adhesion suggests that, by binding to different epitopes of the EGFR extracellular domain, CL4 and cetuximab may have a different effect on integrin-EGFR interaction. We have previously shown that the aptamer binds to the domain IV of EGFR^26^ that is, therefore, plausibly involved in the interaction with integrin αvβ3. However, it would be helpful to localize the sites of the αv and/or β3 and EGFR engaged in the interaction.

In conclusion, we demonstrated that targeting EGFR-integrin αvβ3 interaction by CL4 aptamer, results in inhibition of VM and tumor growth in TNBCs resistant to both erlotinib and cetuximab, raising the possibility of a new aptamer-based therapeutic approach to impair the phenomenon of vasculogenesis in TNBC. This study has the potential to inspire new attempts for finding VM-related strategies in aggressive human cancers.

## Methods

### Aptamers and EGFR inhibitors

2′F-Pyrimidines RNA anti-EGFR CL4 aptamer and the scrambled CL4Sc sequence, used as a negative control, were synthesized by TriLink Biotechnologies and purchased from Tebu-bio srl (Magenta, Milan, Italy).

CL4: 5′GCCUUAGUAACGUGCUUUGAUGUCGAUUCGACAGGAGGC3′;

CL4Sc: 5′UUCGUACCGGGUAGGUUGGCUUGCACAUAGAACGUGUCA3′.

Before each treatment, the aptamers were subjected to a short denaturation-renaturation step as reported^26^. Erlotinib (Cell Signaling Technology Inc., Danvers, MA) and cetuximab (ImClone Systems LLC, Branchburg, NJ) were used as EGFR inhibitors.

### Cell culture

Human breast cancer MDA-MB-231, BT-549, MDA-MB-468, BT-474, BT-20 and MCF-7 cell lines (American Type Culture Collection, ATCC, Manassas, VA) were grown in RPMI-1640 (Invitrogen, Carlsbad, CA, USA) (MDA-MB-231, BT-549 and MDA-MB-468), ATCC HybriCare Medium (BT-474) or Dulbecco’s modified Eagle’s medium (Invitrogen) (BT-20 and MCF-7) supplemented with 10% fetal bovine serum (FBS) (Invitrogen), in 95% air/5% CO2 atmosphere at 37 °C. Growth conditions for mouse NIH3T3 cells were previously reported^26^.

### Western blot and Immunoprecipitation

Cell lysates preparation and Western blots analyses were performed as previously reported^26^. Filters were probed with the indicated primary antibodies: anti-E-cadherin, anti-Vimentin, anti-PDGFRβ, anti-phospho-EGFR (Tyr1068, indicated as pEGFR), anti-EGFR (intracellular), anti-integrin β3 (D7X3 P), anti-integrin αv (D2N5H) (Cell Signaling Technology Inc.); anti-PARP (H-250), anti-α-tubulin (TU-02), anti-vinculin (N-19) (Santa Cruz Biotechnology Inc.); anti-Ku-80 (AHP317; Serotec, Oxford, UK). Densitometric analyses were performed on at least two different expositions to assure the linearity of each acquisition using ImageJ (v1.46r). Blots shown are representative of at least three independent experiments. For immunoprecipitation (IP) with anti-EGFR antibody (Cell Signaling Technology Inc.), 150 μg cell lysates or 300 μg tumor lysates (from 3 individual tumors for each treatment group) were incubated with the antibody at the recommended dilution. For IP with anti-αvβ3 LM609 (Millipore Co., Bedford, MA), 300 μg cell lysates or 1 mg tumor lysates were incubated with 3 μg or 10 μg antibody, respectively. After 2 hours incubation at 4 °C, immunoprecipitation was performed with protein A/G-agarose (Santa Cruz Biotechnology Inc.) overnight at 4 °C.

### Tube formation assay to measure *in vitro* VM of TNBC cells

MDA-MB-231 (9 × 10^5^ cells/well into 6-well plates, 1.6 × 10^5^ cells into 24-well plates) and BT-549 (5 × 10^5^ cells/well into 6-well plates, 8 × 10^4^ cells into 24-well plates) cells in RPMI-1640 containing 2% FBS were seeded into plates pre-coated with Matrigel (BD Biosciences, Franklin Lakes, NJ, 500 μl/well into 6-well plates, 80 μl/well into 24-well plates) and tube formation was analyzed under a phase-contrast microscopy. Complete loops were quantified by a macro made with the ImageJ software. For immunofluorescence analysis of tube formation, MDA-MB-231 (1.6 × 10^5^ cells/well) and BT-549 (8 × 10^4^ cells/well) cells were seeded into 24-well plates containing glass coverslips slips pre-coated with 80 μl Matrigel and analysed as described under Immunofluorescence paragraph.

### Cell adhesion

MDA-MB-231, BT-549 and NIH3T3 (8.0 × 10^4^ cells/well) cells were incubated with 200 nmol/l CL4, 200 nmol/l CL4Sc, 10 μmol/l erlotinib or 100 μg/mL cetuximab at 37 °C or with 10 μg/mL anti-αvβ3 LM609 (Millipore Co.) at 4 °C. After 1-hour incubation, cells were seeded into 96-multiwell plates, previously coated with 5 μg/mL vitronectin (Invitrogen), rinsed three times with Dulbecco’s Phosphate-Buffered Saline (DPBS) and subjected to blocking in 7.5% BSA/DPBS, and allowed to adhere to vitronectin for 1 hour at 37 °C. Subsequently, non-adherent cells were removed with gentle washing in DPBS and adherent cells were stained with 0.1% crystal violet in 25% methanol. Stained cells were lysed in 10% acetic acid solution and absorbance at 595 nm was measured in a microplate reader. All experiments were performed in triplicate wells.

### Cell viability

Cell viability (3.6 × 10^3^ cells/well, 96-well plates) was assessed by CellTiter 96 AQueous One Solution Cell Proliferation Assay (Promega BioSciences Inc., San Luis Obispo, CA) according to the manufacturer’s instructions.

### Cell growth in 3D cultures

MDA-MB-231 cells (7 × 10^4^ cells/well) in RPMI-1640 containing 2% FBS and 2% growth factor-reduced Matrigel were seeded into 6-well plates pre-coated with Matrigel (500 μl per well). After 7 days, cells were analyzed under a phase-contrast microscopy.

### Immunofluorescence

Cells were fixed in 4% PFA/DPBS for 20 minutes at room temperature, permeabilized with 0.2% Triton X-100/DPBS for 5 minutes and then subjected to blocking in 10% FBS/DPBS for 20 minutes at room temperature. For VE-cadherin staining, cells were incubated with anti-VE-cadherin antibody (Cell Signaling Technology Inc.), washed three times in DPBS, and incubated with Alexa Fluor 568 Anti-Rabbit (Invitrogen). For dual staining of EGFR and integrin αvβ3, cells were incubated with anti-EGFR (extracellular, R&D system, Minneapolis, MN) and anti-αvβ3 LM609 (Millipore Co.) antibodies, washed three times in DPBS, and incubated with Alexa Fluor 488 Anti-Goat and Alexa Fluor 568 Anti-Mouse (Invitrogen). Finally, after three washes in DPBS, cells were incubated with 1.5 μM 4′,6-Diamidino- 2-phenylindole (DAPI, Sigma-Aldrich) and mounted with glycerol/DPBS. Samples were visualized on a Zeiss Axioplan fluorescent microscopy and analysed by Axiovision software (VE-cadherin) or Zeiss LSM 510 META confocal microscopy equipped with an oil immersion Plan-Neofluar 63 × 1.40 objective (EGFR, integrin αvβ3).

For tumor tissue staining, 7 μm paraffin sections harvested from CL4Sc group were treated as described under Immunohistochemistry paragraph for antigen retrieval and blocking and then incubated with anti-EGFR (R&D system, 1:100 dilution) and anti-integrin β3 (Cell Signaling Technology Inc.; 1:100 dilution) primary antibodies, and with Alexa Fluor 488 Anti-Goat and Alexa Fluor 568 Anti-Rabbit (Invitrogen, 1:200 dilution) secondary antibodies. DAPI was used to counterstain nuclei. Samples were visualized on Zeiss LSM 700 META confocal microscopy equipped with an oil immersion Plan-Neofluar 63 × 1.40 objective.

### RT-qPCR

RNA extraction and RT-qPCR were performed as previously described^19^. Primers used were: VE-cadherin, Fwd 5′-GCACCAGTTTGGCCAATATA-3′, Rev 5′-GGGTTTTTGCATAATAAGCAGG-3′; PTGIS, Fwd 5′-ATGCCTGCGAGAGACCCTACA-3′, Rev 5′-GCAAGTCACCTCACCTCTCAGTT-3′; CD34, Fwd 5′-TTGACAACAACGGTACTGCTAC-3′, Rev 5′-TGGTGAACACTGTGCTGATTA-3′; SERPINE2, Fwd 5′-GCCATGGTGATGAGATACGG-3′, Rev 5′-GCACTTCAATTTCAGAGGCAT-3′; SLPI, Fwd 5′-TGTGGAAGGCTCTGGAAAG-3′, Rev 5′-TGGCACTCAGGTTTCTTGTATC-3′. The following primers were used for internal control: β-actin, Fwd 5′-CAAGAGATGGCCACGGCTGCT-3′, Rev 5′-TCCTTCTGCATCCTGTCGGCA-3′; Glyceraldehyde-3-phosphate dehydrogenase (GAPDH), Fwd 5′-AAACAGAAGGCAGCTTTACGATG-3′, Rev 5′-AAATGTTCTGATCCAGTAGCG-3′. All experiments were performed in triplicate wells.

### Animal Tumor Models and Treatments

All experimental procedures complied with the European Communities Council directives (2010/63/EU) and national regulations (D.L. 116/92) and were performed in accordance with National Institutes of Health (NIH) recommendations. The present study was approved by the Italian Ministry of Health (authorization number 38/2015-01-28). MDA-MB-231 cells (10 × 10^6^) were re-suspended in 0.1 ml of 1:1 mix of physiological saline and Matrigel and subcutaneously injected into the flank of Female Balb/c nude mice five-week-old and weighing 18–20 g (Charles River, Milan, Italy). Once tumors became palpable (established), approximately 40–50 mm^3^ [volume = 0.5 × long diameter × (short diameter)^2^], nude mice were randomized into 2 groups: CL4Sc and CL4 (6 animals for group). Tumor-bearing animals were treated by caudal vein injection with 1,600 pmol of CL4 and CL4Sc (1.04 mg aptamer/kg). The long and short diameters of the tumors were measured using slide calipers up to 5 days after the last administration, and the body weight was also measured. Tumor growth was also monitored with high-frequency ultrasound system at 14-day treatment (Vevo 2100 equipment; FUJIFILM VisualSonics, Inc., Toronto, Ontario, Canada).

### Fluorescence Molecular Tomography (FMT)

For FMT studies, tumor-bearing mice were maintained on a diet with a purified, alfalfa-free rodent chow for 15 days before fluorescent imaging to minimize fluorescence in the gut. IntegriSense 680 (Perkin Elmer, Inc.), a commercially available small nonpeptide molecule labeled with near-infrared fluorochrome, that specifically binds to integrin αvβ3, was dissolved in DPBS. After 5 days since the last treatments mice were injected via tail vein with IntegriSense 680 (2 nmoles), and allowed to circulate for 24 hours before imaging. All optical imaging experiments were performed with the FMT4000 Quantitative Tomography Imaging System (Perkin Elmer, Inc.). Mice were placed in a biplanar imaging cassette supplied with the instrument and transilluminated with laser light. Resulting transmission and fluorescence patterns were captured with a thermoelectrically cooled CCD camera, and the position and intensity of fluorescence sources were reconstructed in 3D using the TrueQuant software package (Perkin Elmer, Inc.), supplied with the FMT4000. 3D regions of interest (ROIs) were drawn around tumor regions, and a threshold was applied equal to 30% of the maximum value of fluorescence in the adjoining non-tumor area. The total amount (pmoles) of fluorochrome was automatically calculated relative to internal standards generated with known concentrations of the appropriate dye. After *in vivo* imaging, mice were euthanized, tumors were excised, rinsed with saline, blotted dry, and the imaged using 2D fluorescence reflectance mode.

### Immunohistochemistry

After imaging studies three tumors harvested from CL4Sc and CL4 groups were fixed using 10% formalin for 24 hours. Samples were then paraffin-embedded, sectioned, and stained with hematoxylin and eosin (H&E). Tumor tissue sections (7 μm thick) were dewaxed, rehydrated, and then treated with heat-mediated antigen retrieval using 10 mM citrate buffer (pH 6.0) for 15 minutes. Sections were treated with 3% hydrogen peroxide solution for 10 minutes to quench endogenous peroxidase activity. Non-specific binding was prevented by incubation with 5% normal goat serum for 15 minutes. The sections were then incubated with anti-Ki-67 (1:100 dilution), anti-mouse CD31 (Abcam, Cambridge, MA; 1:400 dilution), anti-integrin β3 antibodies (Cell Signaling Technology Inc.; 1:100 dilution), overnight at 4 °C. Antibody binding was detected using horseradish peroxidase-conjugated secondary antibody for 20 minutes at 37 °C. Sections were visualized using diaminobenzidine (DAB) solution counterstained with hematoxylin. PAS staining, H&E staining, and CD31 immunohistochemistry were used to evaluate the presence of VM. Thus, CD31-stained slides were stained by PAS Kit (Sigma-Aldrich) according to manufacturer’s instructions. Slides were observed using light microscopy.

### Statistics

Statistical values were defined using GraphPad Prism version 7.00 for Windows by student’s *t*-test (two variables) or one-way ANOVA followed by Tukey’s multiple comparison test (more than two variables). *P* value < 0.05 was considered significant for all analyses.

## Additional Information

**How to cite this article:** Camorani, S. *et al*. Aptamer-mediated impairment of EGFR-integrin avβ3 complex inhibits vasculogenic mimicry and growth of triple-negative breast cancers. *Sci. Rep.* **7**, 46659; doi: 10.1038/srep46659 (2017).

**Publisher's note:** Springer Nature remains neutral with regard to jurisdictional claims in published maps and institutional affiliations.

## Supplementary Material

Supplementary Figures
