# Supplementary material for: Aptamer-mediated impairment of EGFR-integrin αvβ3 complex inhibits vasculogenic mimicry and growth of triple-negative breast cancers
Source: Sci Rep. 2017 Apr 20;7:46659. doi: 10.1038/srep46659 (PMC5397976; doi:10.1038/srep46659)
Supplement: Supplementary Figures [file srep46659-s1.pdf]

Aptamer-mediated impairment of EGFR-integrin αvβ3 complex inhibits vasculogenic mimicry and growth of triple-negative breast cancers

Simona Camorani[1], Elvira Crescenzi[1], Matteo Gramanzini[2], Monica Fedele[1], Antonella Zannetti[2] & Laura Cerchia[1].

[1]Istituto per l'Endocrinologia e l'Oncologia Sperimentale "G. Salvatore" (IEOS), Consiglio Nazionale delle Ricerche (CNR), Via S. Pansini 5, 80131 Naples, Italy.

[2]Istituto di Biostrutture e Bioimmagini (IBB), Consiglio Nazionale delle Ricerche (CNR), Via T. De Amicis 95, 80145 Naples, Italy.

Correspondence and requests for materials should be addressed to L. C. (email: cerchia@unina.it) or A.Z. (email: antonella.zannetti@ibb.cnr.it)

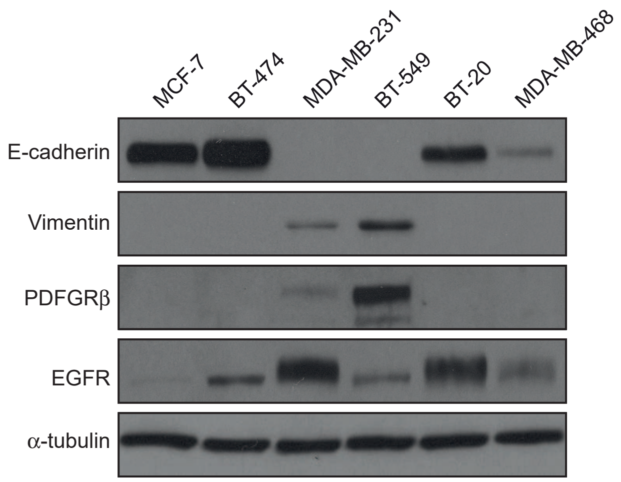

Supplementary Fig. S1. Characterization of breast cancer cells. Lysates from MCF-7 and BT-474 (non-TNBC), MDA-MB-231 and BT-549 (Mesenchymal-like TNBC subtype), BT-20 (Unclassified TNBC subtype) and MDA-MB-468 (Basal-like TNBC subtype) breast cancer cell lines were immunoblotted with anti-E-cadherin, anti-vimentin, anti-PDGFRβ and anti-EGFR antibodies, as indicated. α-tubulin was used as an internal control. TNBC subtypes are according to Lehmann B. D. *et al*.[2]

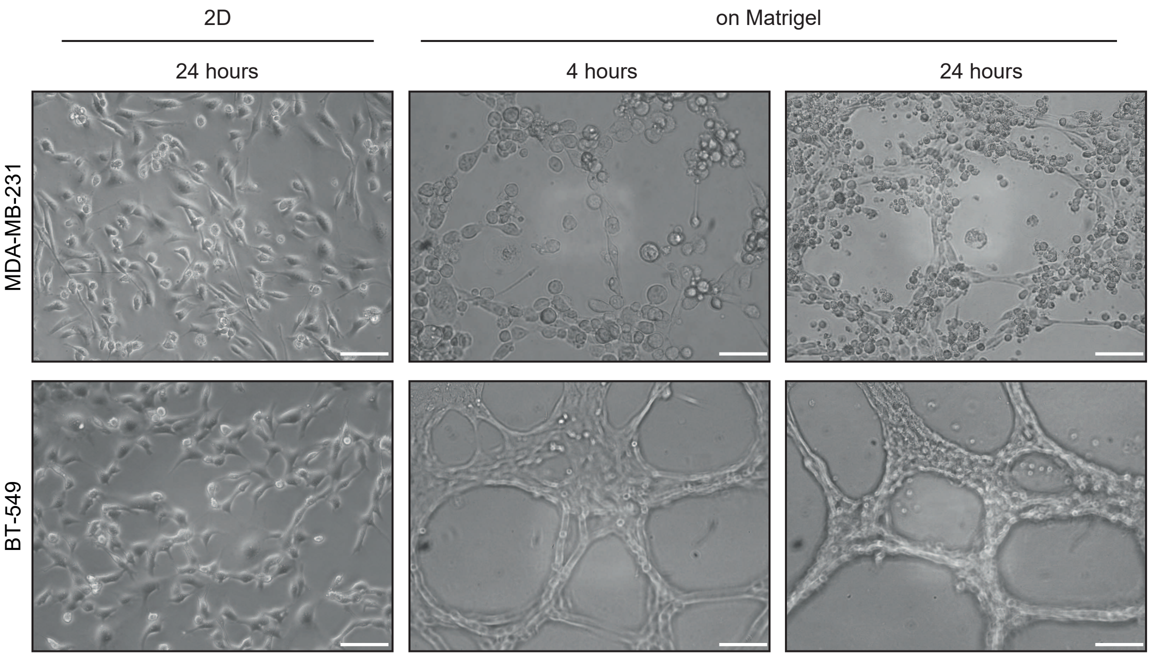

Supplementary Fig. S2. Mesenchymal-like TNBC cells form VM channels on Matrigel. Representative phase-contrast images of MDA-MB-231 cells and BT-549 cells grown in 2D and on Matrigel monolayer for the indicated times. Magnification 20×, scale bar = 100 μm.

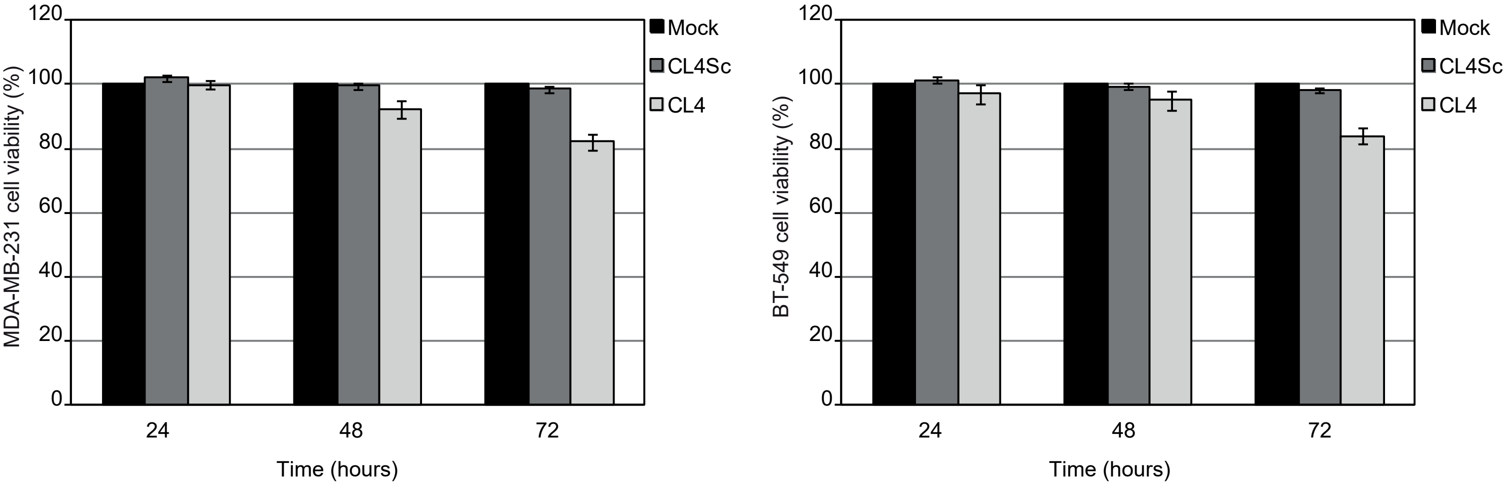

Supplementary Fig. S3. CL4 dosage inhibiting VM does not affect cell viability. MDA-MB-231 (left) and BT-549 (right) cells were mock-treated or treated up to 72 hours with 200 nmol/l CL4 or CL4Sc. Cell viability was analyzed and expressed as percent of viable treated cells with respect to mock-treated cells. The aptamers treatment was renewed each 24 hours. Each determination represents the average of three independent experiments and error bars represent SD.

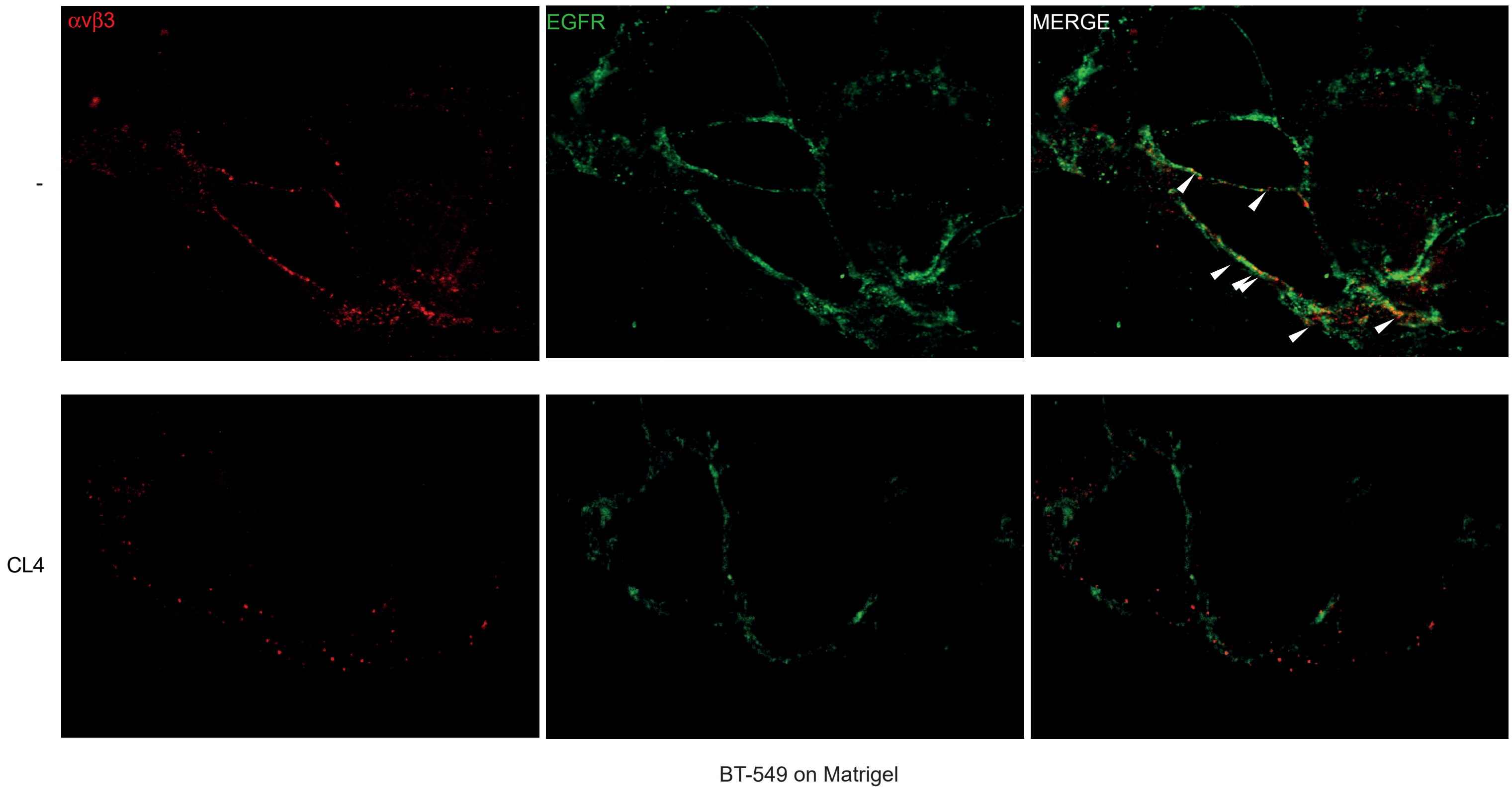

Supplementary Fig. S4. EGFR-integrin αvβ3 co-localization on BT-549 cell membrane. Representative immunofluorescence microscopy images of not permeabilized BT-549 cells grown on Matrigel monolayer in the absence or in the presence of 200 nmol/l CL4 for 24 hours and labelled with anti-αvβ3 LM609 (red) and anti-EGFR (green) antibodies. Co-localization results appear yellow in the merged images. Arrowheads indicate some co-localization points between EGFR and integrin αvβ3. Magnification 63×.

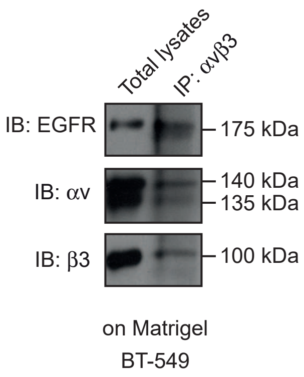

Supplementary Fig. S5. Integrin αvβ3 interacts with EGFR in BT-549 cells. Lysates were prepared from BT-549 cells harvested from Matrigel and total lysates or lysates immunoprecipitated with anti-integrin αvβ3 LM609 antibody were immunoblotted with anti-EGFR, anti-integrin αv and anti-integrin β3 antibodies, as indicated. Molecular weights of indicated proteins are reported. Representative data are shown from one of three independent experiments.

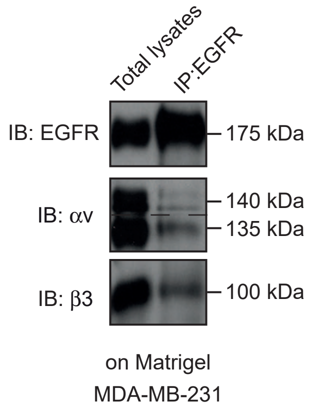

Supplementary Fig. S6. EGFR interacts with integrin αvβ3 in MDA-MB-231 cells. Lysates were prepared from cells harvested from Matrigel and total lysates or lysates immunoprecipitated with anti-EGFR antibody were immunoblotted with the indicated antibodies as reported in the legend to Fig. 3b. Representative data are shown from one of three independent experiments.

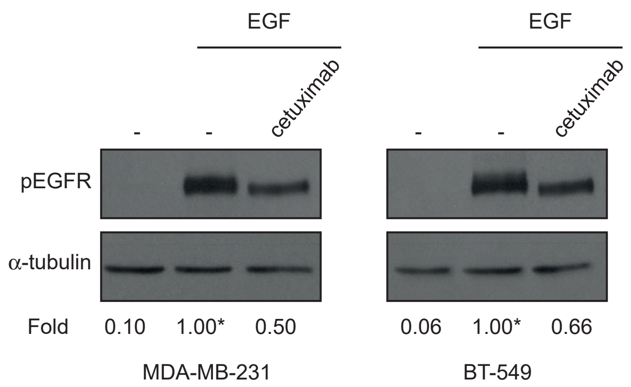

Supplementary Fig. S7. Cetuximab inhibits ligand-induced EGFR phosphorylation in TNBC cells. MDA-MB-231 and BT-549 cells were serum-starved for 18 hours and then left untreated or stimulated with 20 ng/ml EGF in the absence or in the presence of 50 μg/ml cetuximab for 15 minutes. Cell lysates were immunoblotted with anti-pEGFR antibody. Equal loading was confirmed by immunoblot with anti-α-tubulin antibody. Values below the blot indicate pEGFR signal level, normalized to the respective anti-α-tubulin signal level, and reported as relative to EGF stimulated cells, arbitrarily set to 1 (labeled with asterisk).

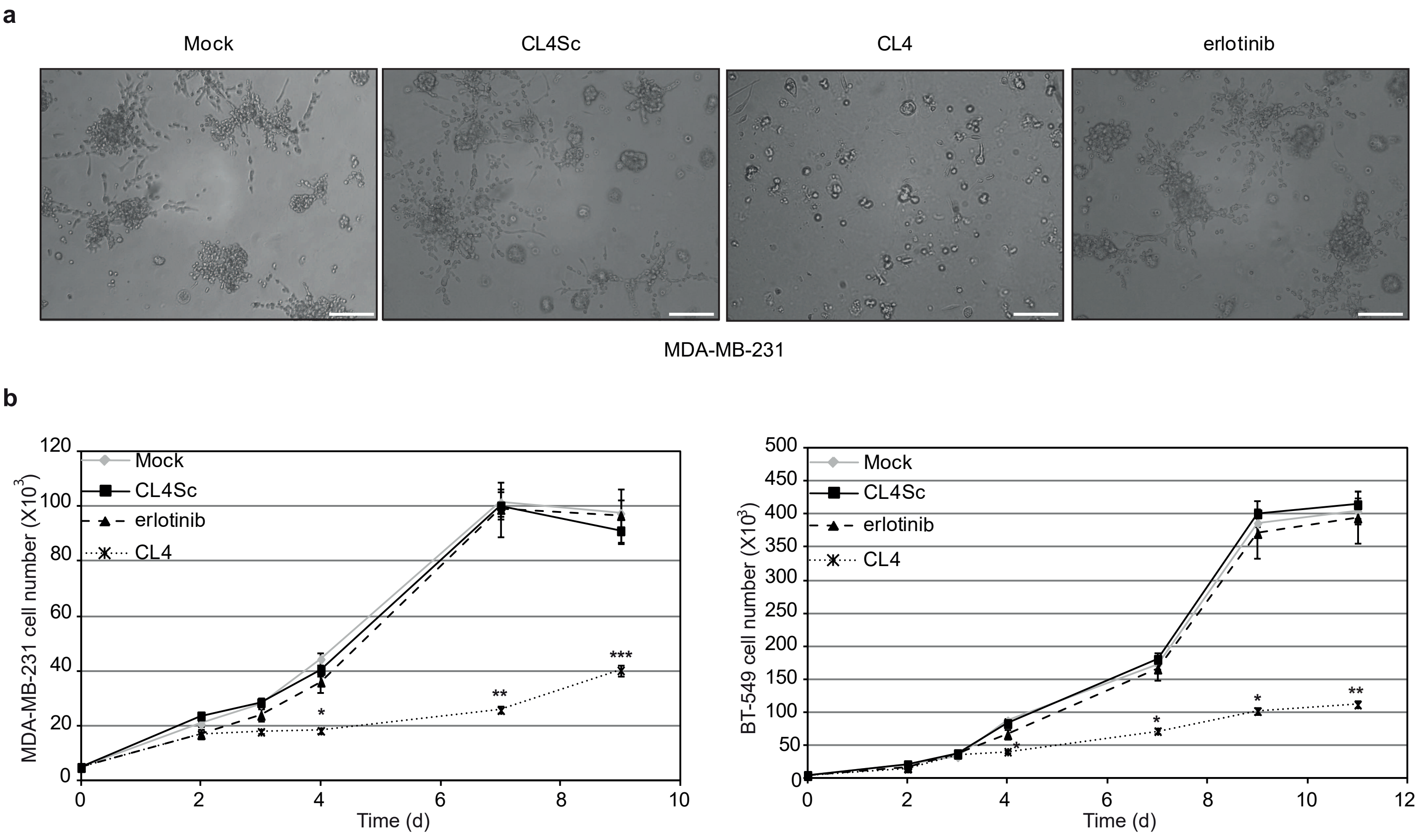

Supplementary Fig. S8. CL4 inhibits growth of TNBC cells in both 2D and 3D culture conditions. (**a**) Representative phase-contrast images of MDA-MB-231 cells grown in Matrigel in the absence or in the presence of 200 nmol/l CL4, CL4Sc or 10 µmol/l erlotinib for 7 days. At least three independent experiments were performed. Scale bar = 200 µm. (**b**) MDA-MB-231 and BT-549 (5×10^3 cells/well in 24-well plates), mock-treated or treated with 200 nmol/l CL4, CL4Sc or 25 µmol/l erlotinib, were counted through the Bürker chamber at the indicated time points. The aptamers treatment was renewed each 24 hours. Growth curves represent the average of three independent experiments and error bars represent SD. ***$P < 0.001$; **$P < 0.01$; *$P < 0.05$ relative to CL4Sc.
